# Altered Dopamine Synaptic Markers in Postmortem Brain of Obese Subjects

**DOI:** 10.3389/fnhum.2017.00386

**Published:** 2017-08-03

**Authors:** Chun Wu, Susanna P. Garamszegi, Xiaobin Xie, Deborah C. Mash

**Affiliations:** ^1^Department of Molecular and Cellular Pharmacology, Miller School of Medicine, University of Miami Miami, FL, United States; ^2^Department of Neurology, Miller School of Medicine, University of Miami Miami, FL, United States

**Keywords:** obesity, BMI, dopamine transporter, tyrosine hydroxylase, dopamine receptor, substantia nigra, striatum

## Abstract

Dopaminergic signaling in the reward pathway in the brain has been shown to play an important role in food intake and the development of obesity. Obese rats release less dopamine (DA) in the nucleus accumbens (NAc) after food intake, and amphetamine stimulated striatal DA release is reduced *in vivo* in obese subjects. These studies suggest that DA hypofunction associated with hedonic dysregulation is involved in the pathophysiology of obesity. To identify brain changes in obesity, quantitative measures of DA synaptic markers were compared in postmortem brain tissues of normal weight and obese subjects over a range of increasing body mass indices (BMI). DA transporter (DAT) numbers in the striatum were compared to the relative expression of DAT, tyrosine hydroxylase (TH) and D2 dopamine receptors (DRD2) in midbrain DA neurons. Radioligand binding assays of [^3^H]WIN35,428 demonstrated that the number of striatal DAT binding sites was inversely correlated with increasing BMI (*r* = −0.47; *p* < 0.01). DAT and TH gene expression were significantly decreased in the somatodendritic compartment of obese subjects (*p* < 0.001), with no significant change in DRD2 compared to normal weight subjects. The reduced density of striatal DAT with corresponding reductions in DAT and TH gene expression in substantia nigra (SN) suggests, that obesity is associated with hypodopaminergic function. A DA reward deficiency syndrome has been suggested to underlie abnormal eating behavior that leads to obesity. Neurobiological changes in presynaptic DA markers demonstrated postmortem in human brain support a link between hedonic DA dysregulation and obesity.

## Introduction

Obesity is one of the leading causes of global deaths. WHO estimates that 39% of adults over 18 years of age are overweight (BMI ≥ 25 kg/m^2^) and, currently 13% meet criteria for obesity (BMI ≥ 30 kg/m^2^)^1^. Recent studies suggest that obesity should be considered as a brain disorder and included in the Diagnostic and Statistical Manual of Mental Disorders (DSM; Devlin, 2007; Volkow and O’Brien, 2007). Food addiction is associated with 4.6 higher BMI units, 8.2% more body fat (Pedram et al., 2013), and is thought to be a valid phenotype of obesity (Davis et al., 2011). Humans can develop food-dependence through learning and habit-formation, and obesity may be considered as a clinical manifestation of food addiction (Volkow and Wise, 2005). Obesity and drug addiction show similarly exaggerated saliency of food or drug reward (Volkow et al., 2013). They share insulin (Daws et al., 2011), leptin (Fulton et al., 2006) and glucagon like peptide-1 (GLP-1; Skibicka, 2013), as common metabolic substrates and underlying neurobiological mechanisms (Kenny, 2011a; Volkow et al., 2012, 2013).

Food intake is regulated by reward signals in the brain, which can override homeostatic pathways by increasing the desire to consume palatable foods (Lutter and Nestler, 2009). Dopamine (DA) neurons in the ventral tegmental area (VTA) are thought to play important roles in motivation and reward (Ikemoto, 2007). Rewarding experiences are accompanied by activation of the nigrostriatal and mesocorticolimbic dopaminergic pathways (Self and Nestler, 1998; Wise, 2009; Ilango et al., 2014). Excitation and inhibition of substantia nigra (SN) DA neurons induce reward and aversion, respectively, and the extent of these effects is similar to those of the mesolimbic VTA DA neurons (Ilango et al., 2014). These results demonstrate that DA neurons of the VTA and the SN contribute to the affective deficits of anhedonia and apathy.

The central importance of the DA transporter (DAT) in regulating dopaminergic neurotransmission has long been demonstrated (Giros et al., 1992). DAT function is important for regulating synaptic levels of the neurotransmitter (Sasaki et al., 2012) and studies in rodents demonstrate decreased cell surface trafficking of the DAT to DA synapses in the nucleus accumbens (NAc) of the striatum in rats fed a high-fat diet (South and Huang, 2008; Cone et al., 2013). Despite the role of dysregulated DA in weight gain, conflicting results have been reported regarding the relationship between BMI and DAT (Chen et al., 2008; Thomsen et al., 2013) and changes in D2DR receptors measured *in vivo* with selective PET radioligands (Wang et al., 2001; Karlsson et al., 2015).

Tyrosine hydroxylase (TH) is the synthetic enzyme that regulates DA concentrations in brain (Daubner et al., 2011). As a major enzyme of catecholamine synthesis, TH is significantly correlated with catecholamine content and neurotransmitter turnover (Bacopoulos and Bhatnagar, 1977; Daubner et al., 2011). Teegarden et al. (2008) showed that exposure to a high-fat diet decreases both TH and DAT expression in midbrain DA neurons, suggesting a deficiency of DA neurotransmission in high-fat rodent models. However, whether DAT or TH gene expression is dysregulated in the condition of human obesity is still unknown. We hypothesized that there is a negative correlation in obesity between BMI and striatal DAT protein levels together with coordinated decreases in presynaptic DA markers of uptake and synthesis. We quantified DAT, D2DR and TH gene expression in the SN with quantitative measures of the number of DAT binding sites in the striatum from obese subjects that came to autopsy over a range of body mass indices (BMI). The present study provides evidence from postmortem human brain that obesity is associated with changes in DA set point that contribute to the intractable cycle of hedonic over-eating.

## Materials and Methods

### Subjects

Postmortem neuropathological specimens were obtained from unaffected control subjects that came to routine autopsy. The biospecimens of brain were provided by the University of Miami Brain Endowment Bank™, an IRB-approved biorepository that holds formalin-fixed, paraffin-embedded and frozen brain tissue annotated with demographic information and clinical variables. Forensic pathologists conducted medicolegal investigations of the deaths (Stephens et al., 2004). The autopsy and toxicology results were reviewed carefully to determine the cause and manner of death. All cases were evaluated for common drugs of abuse and alcohol, and positive urine screens were confirmed by quantitative analysis of blood. There was no gross pathology or neuropathogic changes in brain reported at autopsy, consistent with the lack of any head trauma or history of neurologic disorders prior to death. Drug-free age-matched control subjects (*N* = 40) over a range of BMI were selected from accidental or cardiac sudden deaths with negative urine screens for all common drugs and there was no history of licit or illicit drug use prior to death. All subjects died suddenly without a prolonged agonal state. Since agonal state may affect the RNA expression profile of postmortem brain tissue, care was taken to match subject groups as closely as possible for age, postmortem interval (PMI) and brain pH. Regional samples of postmortem brain were sampled at the level of the ventral striatum and midbrain DA neurons of the SN from frozen coronal blocks based on cytoarchitectural landmarks.

### DA Transporter Binding Assay

DAT binding assays were done as described previously (Mash et al., 2002). Briefly, ventromedial putamen (200 mg) was dissected from cryopreserved brain specimens. Tissues were homogenized in 10 mM Na-Phosphate/0.32 M sucrose buffer with Brinkman Polytron at setting 2.5 for 15 s and centrifuged at 32,000× *g* for 15 min. The membrane pellet was washed once and re-suspended in phosphate-sucrose buffer at a dilution of 1:20 (w/v). 0.5 nM [^3^H]WIN35,428 (0.5 nM, 84Ci/mmol, PerkinElmer) and incubated with 5 mg/well membranes in the presence of increasing concentrations of unlabeled [^3^H]WIN35,428 (0.1 nM–10 μM) for 2 h at 4°C. The reaction was terminated by rapid filtration through 934AH filters and measured by liquid scintillation counting.

### Real-time PCR

Total RNA from 100 mg of frozen SN/case was extracted using RNeasy Lipid Tissue Mini Kit (#74804; Qiagen Inc.) with on column DNase I treatment. RNA concentration was measured with NanoDrop 2000 spectrophotometer and the integrity of total RNA was determined with Agilent 2100 Bioanalyzer. RNA integrity numbers Reverse transcription was performed with High Capacity cDNA Reverse Transcription Kit (#4368813) using random primers from Applied Biosystems. Expression levels of target genes were measured with TaqMan assays on 7900HT Real Time PCR (ABI). TaqMan probes and primers were purchased from Applied Biosystems: DAT1 [SLC6A3] (Hs00997374_m1), TH (Hs00165941_m1), D2 dopamine receptors (DRD2; Hs01024460_m1) ACTB (Hs99999903_m1), B2M (Hs99999907_m1) and PPIA (Hs99999904_m1) were used as housekeeping genes for normalization.

### Statistical Analysis

Standardized DAT, DRD2, or TH gene expression was calculated using the comparative Ct method with DataAssist™ Software (ABI, Foster City, CA, USA). Relative target gene expression was normalized with the geometric mean of the expression levels of three “housekeeping” genes. Estimation of total DAT binding sites for [^3^H]WIN35,428 was done by using non-linear curve fitting software LIGAND (Biosoft, KELL v.6.0, Cambridge, UK) and PRISM (v.6.0, GraphPad, San Diego, CA, USA). Correlation and linear regression between BMI and DAT/TH/DRD2 expression levels were analyzed by GraphPad Prism (v.6.0) and JMP (v.13, SAS Institute).

## Results

Each case selected for this study was evaluated for cause and manner of death, forensic toxicology and pathologic investigation. None of these cases tested positive for any neuroactive drug or metabolite, which would affect the DA reward system. Table 1 shows demographic characteristics of the cohorts used for DAT binding in the striatum and qPCR measures of gene expression in the midbrain SN. Striatal samples had a range of BMI values from 20.1 kg/m^2^ to 39.1 kg/m^2^ (*N* = 40). The BMI range for the SN was 18.1–43.8 kg/m^2^ with 13 in the normal weight range (BMI ≤ 25 kg/m^2^) and 16 out of a total of 40 that were morbidly obese (BMI ≥ 30 kg/m^2^). Midbrain samples of DA neurons were included for gene expression analysis if the RIN values were ≥6.0. We substituted additional cases for the gene expression analysis to balance the sample size of both cohorts across normal weight, overweight and obese groups to closely match samples based on demographic variables (Table 1; *N* = 40).

**Table 1:** Demographic characteristics of normal weight and obese subjects.

Region	Striatum	Substantia nigra
**Number of subjects**	*N* = 40	*N* = 40
Normal weight	*N* = 16	*N* = 13
Overweight	*N* = 12	*N* = 11
Obese	*N* = 12	*N* = 16
**BMI**		
Mean	27.3 ± 4.8	29.4 ± 6.7
Range	20.1–39.1	18.3–43.8
**Age**	36.3 ± 14.0	40.6 ± 14.1
**PMI**	16.6 ± 5.6	16.7 ± 6.3
**Race**		
Caucasian	*N* = 29	*N* = 31
African-American	*N* = 11	*N* = 9
**Gender**		
Male	*N* = 33	*N* = 34
Female	*N* = 7	*N* = 6
**RIN**	N/A	7.6 ± 0.9

### Negative Correlation between Striatal DAT Binding Sites and BMI

**Figure 1:**
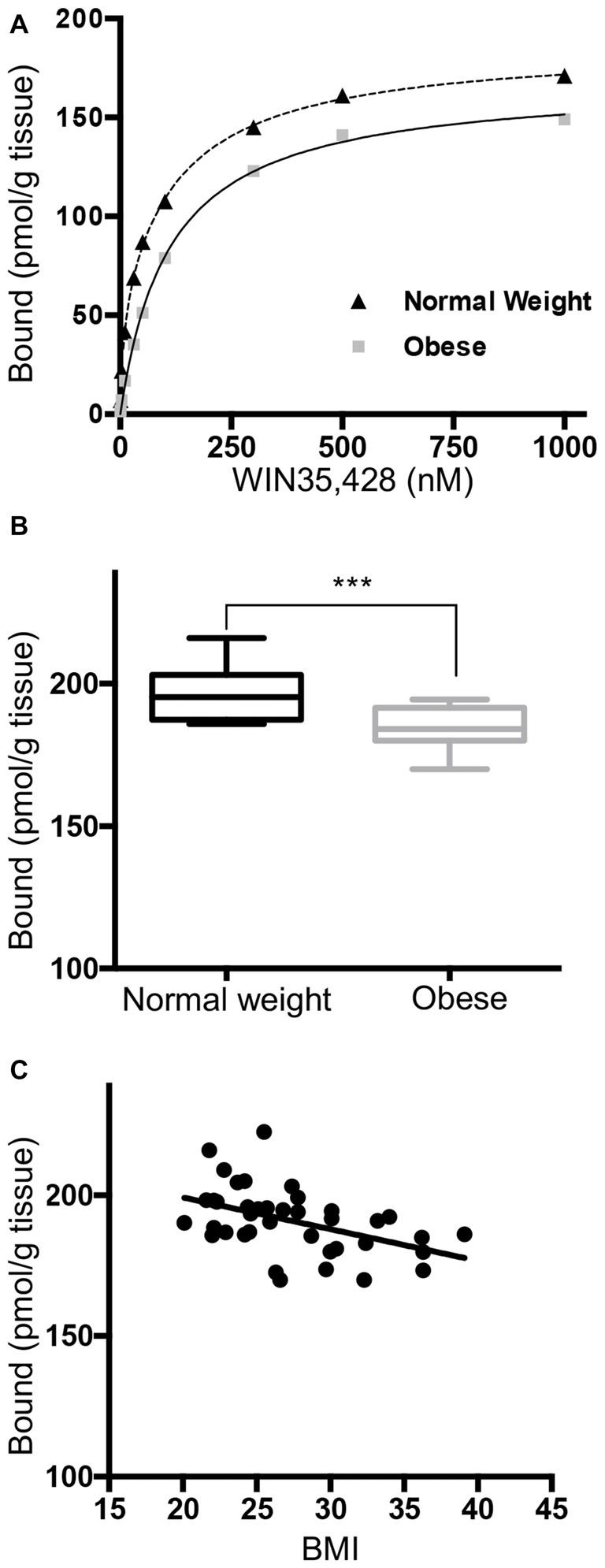
Striatal [^3^H]WIN35,428 binding shows a negative correlation between dopamine transporter (DAT) density and body mass indices (BMI). **(A)** Representative saturation binding of [^3^H]WIN35,428 to the DAT in a normal weight control (24.2 kg/m^2^) and an obese subject (36.2 kg/m^2^). Radiolabeled [^3^H]WIN35,428 (0.5 nM) was incubated with striatal membranes in the presence of increasing concentrations of unlabeled WIN35,428. Estimation of total DAT binding sites was done by using non-linear curve fitting software LIGAND. **(B)** DAT binding sites (pmol/g tissue) in ventral striatum were significantly decreased in obese subjects (BMI ≥ 30 kg/m^2^, *n* = 12) compared to normal weight controls. **(C)** DAT binding sites were inversely correlated with BMI values (20.1–39.1; Pearson *r* = −0.47, *p* = 0.0025, *n* = 40). Student’s *t*-test was used to compare normal weight and obese subjects, ***p < 0.001.

DAT density in the striatum of postmortem brains was quantified by assays of [^3^H]WIN35,428 binding sites. Saturation analysis of the total amount of ligand bound confirmed a significantly lower number of DAT binding sites in the obese group (Figures 1A,B, *p* < 0.001) compared to normal weight subjects. The overall maximum binding site density was negatively correlated with BMI by linear regression analysis (Figure 1C, Pearson *r* = −0.47, *p* = 0.0025). Multiple regression analysis (Supplementary Table S1) with adjustment for age, PMI, gender and race confirmed the significant effect of BMI (*p* = 0.0046). The results demonstrate that PMI, gender and race did not affect the number of DAT binding sites in this study.

### DAT mRNA was Down-Regulated in the Substantia Nigra of Obese Subjects

**Figure 2:**
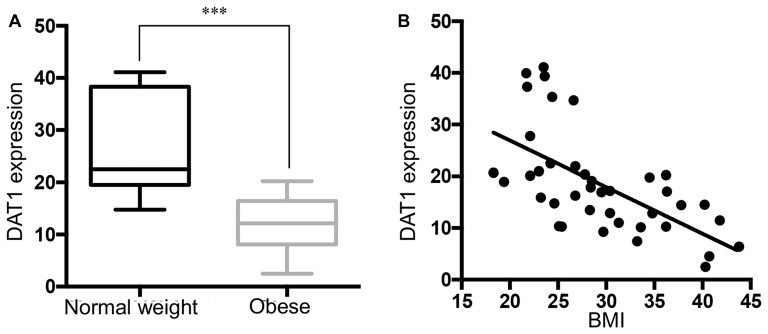
Reduced DAT expression in midbrain of obese subjects. **(A)** DAT mRNA expression in normal weight (BMI < 25 kg/m^2^, *n* = 13) and obese subjects (BMI ≥ 30 kg/m^2^, *n* = 16). **(B)** A scatter plot of BMI vs. normalized DAT gene expression showed a negative correlation (*n* = 40, Pearson *r* = −0.615, *p* < 0.0001). Student’s *t*-test of DAT expression in normal weight and obese subjects, ***p < 0.001.

We measured DAT mRNA expression levels across a range of BMI values in the DA cell body field of the midbrain. Unpaired Student *t* test of the sample means revealed a significant decrease of DAT mRNA in the obese group (Figure 2A, *p* < 0.001). Linear regression (Figure 2B) showed a significant negative correlation between DAT and BMI (*n* = 40, Pearson *r* = −0.615, *p* < 0.001). Multiple regression analysis (Supplementary Table S1) of age, race, PMI, gender and RIN values confirmed that BMI value was the only significant variable (*p* < 0.001). This analysis demonstrates that there was no effect of gender or any of the other demographic variables for the cohorts tested on the qPCR measures of gene expression obtained in this study.

### Negative Correlation between TH mRNA and BMI

**Figure 3:**
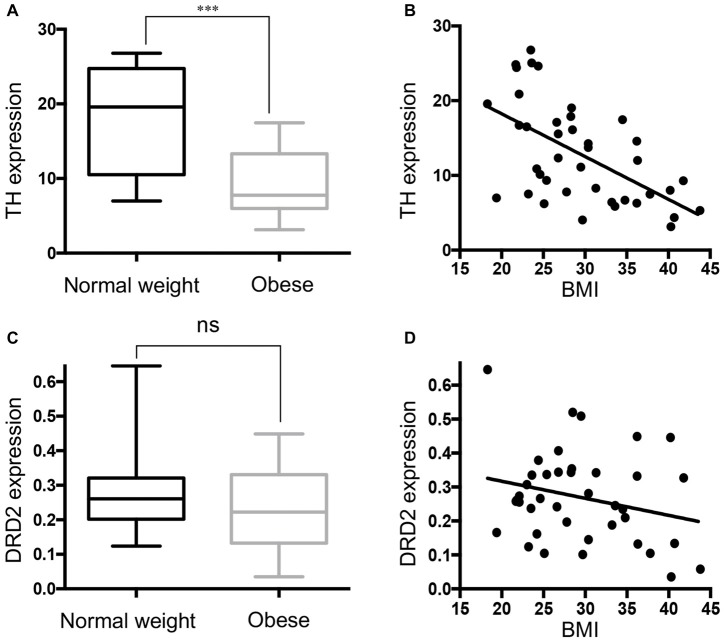
Tyrosine hydroxylase (TH) expression is downregulated in the midbrain of obese subjects. **(A)** TH gene expression in individual cases of normal weight controls (BMI < 25 kg/m^2^, *n* = 13) and obese subjects (BMI ≥ 30 kg/m^2^, *n* = 16). **(B)** A scatter plot of the normalized TH gene expression showed a significant negative correlation with increasing BMI values (*n* = 40, Pearson *r* = −0.575, *p* < 0.001). **(C)** D2 dopamine receptor (DRD2) expression in individual cases of normal weight controls (*n* = 13) and obese subjects (*n* = 16). **(D)** A scatter plot of the normalized DRD2 gene expression and BMI (*n* = 40). Student’s *t*-test was used to compare normal weight and obese subjects; ***p < 0.001.

Figure 3A shows that TH gene expression was significantly down-regulated in obese subjects, in keeping with decreased DAT mRNA. Correlation analysis showed that TH gene expression was negatively correlated with BMI in the SN (Figure 3B). Multivariate regression demonstrated the association of TH with BMI (*p* < 0.001), but not with age, race, PMI, gender and RIN. The results demonstrate that there was no significant change (*p* = 0.1156) in DRD2 expression in the SN in the midbrain of obese subjects that came to autopsy (Figures 3C,D). DAT gene expression was correlated with TH gene expression levels (*p* < 0.0001), but not with DRD2 expression (*p* = 0.1159; Supplementary Figure S1).

## Discussion

Here, we report our findings on the neurobiological changes in DA synaptic markers that are associated with obesity. Our studies in postmortem human brain demonstrate a reduced number of striatal DAT binding sites associated with lower DAT and TH gene expression in midbrain DA neurons. To the best of our knowledge, this report is the first postmortem human brain study to support a lower DA set point in the brain of obese subjects that came to autopsy.

Body weight is determined by both metabolic and hedonic mechanisms that crosstalk with each other to maintain a homeostatic energy balance. Alterations in the food reward system that leads to excessive caloric ingestion and obesity is explained in part on the basis of two prevailing hypotheses related to the “liking” and “wanting” aspects of the DA reward system. We demonstrated overall reductions in DA synaptic markers DAT and TH, that were negatively correlated with BMI values that ranged from overweight to extreme obesity. The results of our study support the hypothesis that a hyporesponsiveness of DA leads individuals to engage in addictive overeating to compensate for the underarousal that drives a food addiction (Volkow and Wise, 2005; Morin et al., 2017).

Studies in animal models suggest that high calorie food may be as addictive as cocaine and heroin, since compulsive eating habits are similar to the intractable pattern of drug addiction (Johnson and Kenny, 2010). Palatable food or drugs are addictive, because they increase DA levels in the reward centers of the brain. Consumption of palatable food is compulsive and triggers down-regulation of striatal D2 receptors (D2Rs) in obese rats (Johnson and Kenny, 2010), consistent with lower striatal D2Rs occupancies reported in obese humans (Volkow et al., 2008). These observations suggest that obesity is a reward deficiency syndrome, that shares common neurochemical defects in DA signaling similar to what has been reported in chronic drug abusers (Kenny et al., 2013). Such parallels have generated significant interest in understanding the shared vulnerabilities and trajectories between drug addiction and the overeating that leads to obesity.

The DA transport regulates DA levels in the brain by controlling DA reuptake from the synaptic cleft (Zahniser and Doolen, 2001). Rodents fed a high-fat diet have reduced striatal DAT and impairment of nigrostriatal DA function (Huang et al., 2006; South and Huang, 2008), which are in agreement with our findings in postmortem human brain. Chen et al. (2008) demonstrated a negative correlation between striatal DAT and BMI values in SPECT imaging of healthy volunteers. The DAT is regulated by psychostimulants in response to elevated synaptic DA (Mash et al., 2002; for review, Zahniser and Doolen, 2001). DAT regulation of synaptic DA is one of the hedonic mechanisms regulated by “pleasure” signals that drive intractable drug-taking. We speculate based on the results of our study, that long-lasting counteradaptations in DAT function may underlie the change in DA homeostasis that makes a person vulnerable to overeating to satisfy their cravings for food reward (Morin et al., 2017).

Compensatory changes in the DA synthesis marker TH and DA reuptake correlate with lower DA output and signaling in obesity, but it is unknown how they contribute to food intake regulation. Recent studies have demonstrated direct leptin effects on DA neuron function, providing a mechanism by which peripheral hormones influence behavior and emphasize a role for DA in the neural control over food intake (DiLeone, 2009). Leptin deficient *ob/ob* mice have lower levels of TH in brain (Fulton et al., 2006; Kenny, 2011b). Peripheral leptin replacement elevates TH levels in the VTA and ventral striatum of ob/ob mice (Fulton et al., 2006). These studies demonstrate that leptin directly targets TH expression in DAergic neurons, a mechanism by which leptin may directly influence brain reward circuitry. Consistent with these findings, we showed that TH gene expression was significantly down-regulated in midbrain DA neurons of obese humans. Because leptin action in brain reduces food intake and body weight by regulating DA signaling, adaptive changes in DA synaptic markers may be causally related to the pathogenesis of obesity in humans. Reduced basal DA signaling may drive the tendency to increase food intake, because it is related to the hedonic attractiveness of food despite the opposing effects of leptin signals that promote satiety.

Deficits in insulin function disrupt the DA system and increase the animal’s propensity to over consume palatable high-fat food (Daws et al., 2011). The precise mechanism underlying lower DA transport function in obesity is not fully understood. One possibility is that insulin signaling in the brain is deficient, since insulin levels in the CNS are negatively correlated with BMI in humans (Tschritter et al., 2009). Intraventricular insulin delivery to the brain increases DAT mRNA expression in rat midbrain DA neurons (Figlewicz et al., 1994). More recent studies demonstrate that insulin regulates the human DAT through an AKT and GSK-3 dependent pathway (Williams et al., 2007; Speed et al., 2011). Insulin signaling in midbrain DA neurons is necessary for DA synthesis and release (Kamei and Ohsawa, 1996; Murzi et al., 1996; Kenny, 2011a). In rats, a high-fat diet rapidly causes insulin signaling deficiencies and corresponding reductions in cell surface DAT expression in nigrostriatal neurons (Speed et al., 2011). The disruption of brain insulin signaling (insulin resistance) might confer risk for “food use” disorders (Daws et al., 2011) through coordinated dysregulation of DA reward. These studies underscore a possible link between changes in DA transport and insulin sensitivity, as an underlying neurobiological function that contributes to obesity as a clinical manifestation of food addiction (Daws et al., 2011).

Presynaptic DRD2 mRNA expression was not significantly different in midbrain DA neurons of obese subjects compared to normal weight subjects. D2 autoreceptors located on the dendrites and cell bodies of DA neurons decrease neuronal excitability, which leads to reduced DA synthesis and vesicular storage (Baik, 2013). Our results suggest that these functions mediated by D2 autoreceptors may be less affected in the condition of obesity than the decreases in DAT and TH gene expression. Consistent with this hypothesis, our recent RNA-Seq analysis of postmortem brain from obese subjects showed significant decreases in DRD2 gene expression in the caudate (data not shown), which agree with *in vivo* PET imaging studies of D2DR receptor occupancy in the striatum of obese subjects (Wang et al., 2001). These results suggest that down-regulation of DRD2 may be associated with postsynaptic receptors on striatal neurons that receive DA projections from midbrain neurons.

Animal studies demonstrate a causal relationship between defects in the reward system and the onset and maintenance of obesity. Laboratory rats fed with a cafeteria diet for 15 weeks develop obesity, which is associated with reduced extracellular DA (Geiger et al., 2009), suggesting that diet-induced alterations in food reward may contribute to compulsive overeating. The food addiction concept compares binge eating of rewarding food items due to a reward deficiency to the addictive process of reward tolerance. Our studies in postmortem human brain demonstrate that DA synaptic markers are dysregulated in the nigrostriatal pathway of obese subjects, providing evidence for defective hedonic signaling in response to palatable foods that may drive pathological overeating. Although body weight has a genetic basis, the exposure to high caloric food may cause allostatic changes in DA signaling pathways that lead to obesity. We suggest that dysregulated DA signaling in the brain’s reward pathways contributes to an intractable cycle of excessive food intake that overrides homeostatic metabolism.

## Author Contributions

SPG, XX conducted experiments. CW, SPG and DCM conducted data analysis; wrote the manuscript and contributed to the interpretation of results. All the authors approved the manuscript to be published.

## Conflict of Interest Statement

The authors declare that the research was conducted in the absence of any commercial or financial relationships that could be construed as a potential conflict of interest.

## Footnotes

^1^www.who.int

## Supplementary Material

FIGURE S1 Correlation analyses of dopamine transporter (DAT), tyrosine hydroxylase (TH) and D2 dopamine receptors (DRD2) gene expression in the substantia nigra (SN). **(A)** A scatter plot of the normalized DAT and TH gene expression showed a significant positive correlation (*p* < 0.0001). **(B)** Correlation analysis of the normalized DAT and DRD2 gene expression levels in SN (*p* = 0.1159).
