# Supplementary material for: Altered Dopamine Synaptic Markers in Postmortem Brain of Obese Subjects
Source: Front Hum Neurosci. 2017 Aug 3;11:386. doi: 10.3389/fnhum.2017.00386 (PMC5541030; doi:10.3389/fnhum.2017.00386)
Supplement: Supplementary file 1 [file Table_1.docx]

**Supplementary Tables**

| **Table S1. Multiple Regression Analysis Based on Demography and Agonal State Variables** | | | |
| --- | --- | --- | --- |
| DAT binding sites | | | |
| Variable | Sum of Squares | F Ratio | p-value |
| BMI | 1044.2224 | 9.196 | *0.0046 |
| Age | 27.3383 | 0.2408 | 0.6268 |
| Race | 21.9125 | 0.193 | 0.6632 |
| PMI | 117.5075 | 1.0348 | 0.3162 |
| Gender | 2.8343 | 0.025 | 0.8754 |
| DAT gene expression | | | |
| Variable | Sum of Squares | F Ratio | p-value |
| BMI | 1458.9796 | 21.6828 | *<0.0001 |
| Age | 1.6031 | 0.0238 | 0.8783 |
| Race | 55.6639 | 0.8273 | 0.3697 |
| PMI | 11.6382 | 0.173 | 0.6802 |
| Gender | 10.4151 | 0.1548 | 0.6965 |
| RIN | 29.8808 | 0.4441 | 0.5098 |
| TH gene expression | | | |
| Variable | Sum of Squares | F Ratio | p-value |
| BMI | 605.22323 | 18.5751 | *0.0001 |
| Age | 18.37801 | 0.564 | 0.458 |
| Race | 17.0311 | 0.5227 | 0.4748 |
| PMI | 21.01739 | 0.6451 | 0.4276 |
| Gender | 29.70397 | 0.9117 | 0.3466 |
| RIN | 14.47873 | 0.4444 | 0.5097 |

*p-value < 0.005
