# Supplementary figures and images for: Altered Dopamine Synaptic Markers in Postmortem Brain of Obese Subjects
Source: Front Hum Neurosci. 2017 Aug 3;11:386. doi: 10.3389/fnhum.2017.00386 (PMC5541030; doi:10.3389/fnhum.2017.00386)

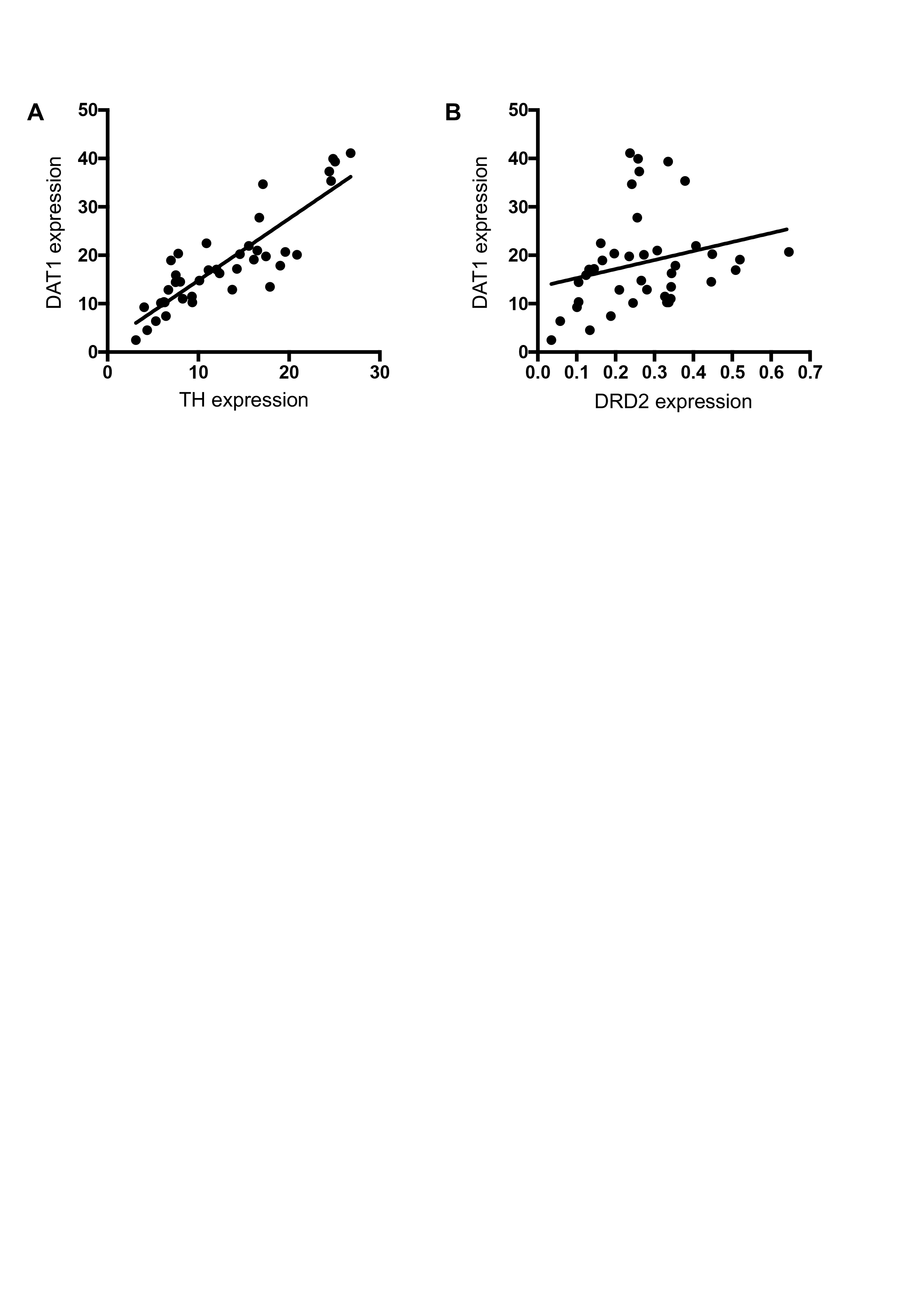

Supplement: FIGURE S1 — Correlation analyses of dopamine transporter (DAT), tyrosine hydroxylase (TH) and D2 dopamine receptors (DRD2) gene expression in the substantia nigra (SN). (A) A scatter plot of the normalized DAT and TH gene expression showed a significant positive correlation (p < 0.0001). (B) Correlation analysis of the normalized DAT and DRD2 gene expression levels in SN (p = 0.1159). [file Image_1.tif]
